# Clinical and Dermoscopic Comparison of the Efficacy and Safety of 5% Fluorouracil Topical Cream and 1% Niacinamide Topical Gel in the Treatment of Actinic Keratosis: A Randomized Controlled Trial

**DOI:** 10.1111/jocd.16676

**Published:** 2024-11-26

**Authors:** Nazila Poostiyan, Mahbube Barati, Zabiholah Shahmoradi, Mina Saber

**Affiliations:** ^1^ Department of Dermatology, Skin Diseases and Leishmaniasis Research Center, School of Medicine Isfahan University of Medical Sciences Isfahan Iran

**Keywords:** actinic keratosis, dermoscopy, fluorouracil, niacinamide, treatments

## Abstract

**Background:**

Actinic keratosis (AK) is a common skin condition treated by dermatologists; however, the effectiveness, superiority, and potential side effects of current treatment protocols are still debated.

**Aim:**

This study aimed to compare the effectiveness and safety of 5% fluorouracil topical cream and 1% niacinamide topical gel in patients with AK.

**Methods:**

In a randomized clinical trial, 26 patients with 95 AK lesions were assigned to receive either 5% fluorouracil topical cream twice daily for 4 weeks or 1% niacinamide topical gel twice daily for 3 months. Photography and dermoscopy before and after treatment were used to evaluate the outcomes.

**Results:**

The study included 26 patients who underwent randomization and treatment. Analysis of the improvement response after treatment through photography and dermoscopy scores, as well as patients' perspectives, showed that the fluorouracil group had significantly better outcomes than the niacinamide group. However, treatment complications including burning, itching, and erythema were significantly more frequent in the fluorouracil group than in the niacinamide group.

**Conclusions:**

Although 5% fluorouracil cream is more effective than 1% niacinamide gel in treating AK lesions, it is also associated with more frequent side effects.

## 1 Introduction

Actinic keratosis (AK) is keratinocyte dysplasia or intra‐epithelial neoplasm and is white people's most common premalignant skin condition. Actinic keratosis is among the most common conditions for patients to consult with dermatologists and has a prevalence of 37.5% among whites 50 years of age or older [1, 2]. The disease is also more prominent in men, with an age‐related linear increase [2]. These lesions usually present as erythematous and keratotic or scaling plaques with a rough, sandpaper‐like surface on chronically sun‐exposed areas, such as the face, ears, arms, and dorsal hands [1, 2]. The main constitutional and exposure risk factors include chronic exposure to ultraviolet radiation, use of sunbeds, advanced age (up to 80% of adults aged > 60 years are affected), male sex, Fitzpatrick skin type I–II, prolonged immunosuppression, and previous history of AK and non‐melanoma skin cancer [1, 2, 3]. Actinic keratosis may progress into squamous‐cell cancer if neglected. If multiple AKs are present and accompanied by signs of chronic actinic damage or field cancerization, the risk of malignant conversion increases rapidly [3]. Therefore, prompt diagnosis and effective treatment are vital to avoid long‐term morbidity and mortality [4].

Dermoscopy is a non‐invasive examination that improves the clinical diagnosis and early and efficient management decisions for skin lesions. In addition, a dermoscopic examination contributes to its treatment through the preprocedural assessment of lesion surface boundaries, the monitoring of the effects of local therapies, and the long‐term monitoring of patients [5, 6].

One of the most widely utilized topical treatments for both lesion‐directed and field‐directed therapy of AK is Fluorouracil (5‐FU) due to its effectiveness, simplicity of administration, and cost‐efficiency. 5‐FU is an antimetabolite that inhibits thymidine synthesis, leading to faulty DNA replication and cell death, particularly in rapidly dividing cells, such as abnormal keratinocytes found in actinic keratosis lesions [2, 3]. However, topical 5‐FU therapy is also linked to unfavorable local skin reactions, leading to a local inflammatory response associated with its mode of action [6, 7, 8]. Niacinamide, a type of vitamin B, is a low‐cost, scientifically supported oral treatment option for AK, squamous cell carcinomas, basal cell carcinomas, and bullous pemphigoid. Despite having a good safety record and being reasonably priced, niacinamide is still being integrated into clinical practice, and like many over‐the‐counter vitamins, it has encountered some challenges. Its function in chemoprophylaxis is believed to repair DNA damage and lessen immunosuppression brought on by UV radiation [9, 10]. In recent years, understanding of the pharmacokinetics of topical niacinamide has grown. In ex vivo human skin, niacinamide and its lipophilic analog methyl nicotinate were both similarly absorbed, but niacinamide's in vivo dermal transport was enhanced by a binary vehicle made of propylene glycol and linolenic acid [11, 12]. Although biologically reasonable, niacinamide's effect in the chemoprophylaxis of melanocytic malignancies and other dermatological conditions such as AK has to be further studied in clinical studies [13]. In the current study, we compared the therapeutic effects of 5% fluorouracil with 1% niacinamide in treating AK.

## 2 Materials and Methods

### 2.1 Study Design and Participants

The current study was a randomized, open‐label trial conducted at the University‐affiliated Dermatology Clinics from June 2021 to December 2023. Before treatment, all participating patients received a thorough explanation regarding the treatment duration, follow‐up procedures, and potential side effects. Each patient was required to sign a written consent form. The informed consent and study protocol were approved by the regional Research Ethics Committee.

Patients older than 30 years with the clinical and dermoscopic diagnosis of AK in the head and neck area were eligible for participation. Patients were excluded in cases of suspicion of cancer in the target area, allergy to trial drugs, pregnancy/breast‐feeding, and patients who received previous treatments for AK in the target area, such as cryotherapy, or had used topical medication in the past month, or systemic retinoids or systemic immunosuppressant drugs within the past three months before inclusion. Patients who did not attend follow‐up visits, did not adhere to the study's treatment protocol, took other systemic drugs during the study, were pregnant, or experienced drug‐induced hypersensitivity were excluded from the final analysis.

### 2.2 Treatment Protocol

Patients were randomly divided into two groups to receive either 5% fluorouracil cream or 1% niacinamide gel. Randomization was performed by assigning a random number to each patient by Microsoft Excel function. All AK lesions in each patient were evaluated, regardless of the number of lesions in a treatment group.

In the fluorouracil group, patients were treated with 5% topical cream twice daily for 4 weeks in the affected areas; in the niacinamide group, patients were treated with topical 1% gel twice daily for 12 weeks. All participants received education about skin cancer, sun protection, and the use of a similar sunscreen with a sun protection factor of 50^+^.

### 2.3 Assessments

Treatment response was evaluated through photography by a single‐blinded photographer using a fixed camera setting, as well as dermoscopic evaluation by a single‐blinded dermatologist using a dermatoscope (Handyscope Photo Finder System GmbH Bad Brinbach, Germany). Each patient had two sets of photos taken: one at baseline and another two weeks after the end of treatment (four weeks for 5FU and three months for niacinamide). Post‐treatment photography was concluded two weeks after the end of the treatment period to minimize any inflammation caused by medication. Clinical and dermoscopic photographic assessments were performed by two blinded dermatologists before and after treatment. Each lesion was assigned a score: mild (0%–25% improvement), moderate (25%–50% improvement), good (50%–75% improvement), and excellent (75%–100% improvement).

Description of the presence or absence of classical dermoscopic features of AK classification for each lesion of patients included: rhomboidal pattern, annular granular pattern, jelly sign, star‐like appearance, white/yellowish scales, red pseudo‐network, inner gray halo, white circle sign, rosette, double white clod, one white clod, dermoscopic horn, linear wavy vessels. Classification and scoring of dermoscopic features of AK were defined as 0 for negative, +0.5 for faint, +1 for positive, and + 2 for strongly positive.

Patient treatment satisfaction with their treatments was evaluated using a score ranging from 1 to 10, where 10 represents the highest satisfaction and 1 indicates the lowest.

The adverse effects of medication were evaluated through history taking and physical examination. Patients were asked about any symptoms such as pruritus, burning, and pain. During physical examination, erythema, erosion, and ulceration were assessed. The side effects were scored as 1 (mild), 2 (moderate), and 3 (severe).

### 2.4 Statistical Analysis

Following data collection, data were entered into SPSS Version 23. Descriptive statistics were reported with mean and standard deviation (SD), median and interquartile range (IQR), or frequency and percentage (%). The variables were compared between the two groups using Chi‐Square/Fisher's exact test for categorical variables, Student's *t*‐test for parametric variables, and Mann–Whitney *U*‐test for nonparametric variables. The changes from the before to after of treatment within each group were tested using the Wilcoxon signed‐rank test for nonparametric variables and paired *t*‐test for parametric variables. Also, a repeated measures test was used to compare changes before and after treatment between the two groups. A *p*‐value of less than 0.05 was considered statistically significant.

## 3 Results

**FIGURE 1:**
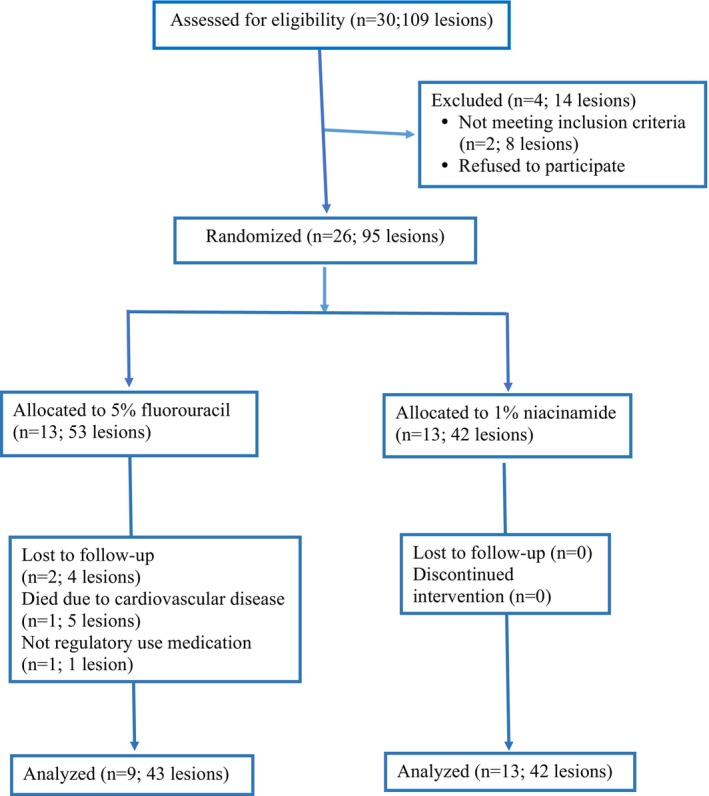
CONSORT chart of the study.

30 patients with AK were evaluated at the beginning of the study, 2 declined to participate, and 2 were excluded. 26 patients with 95 AK lesions were enrolled in the study. The trial was completed by 13 patients (43 lesions) in niacinamide and 9 patients (42 lesions) in the 5FU group respectively (Figure 1).

The majority of patients were male (*n* = 18; 81.8%), and the average age was 67.37 ± 10.76 (range 45–90). The demographic and clinical characteristics of the patients are shown in Table 1. As demonstrated, there was no significant difference regarding age, gender, occupation, skin type, and underlying diseases between the two groups.

**TABLE 1:** Demographic and clinical characteristics of the patients.

Characteristics	Total *N* = 22	Treatment		*p*
		Fluorouracil; *n* = 9	Niacinamide; *n* = 13	
Age (years); mean ± standard deviation	67.37 ± 10.76	62.88 ± 11.53	70.75 ± 9.21	0.111
**Gender; *n* (%)**				
Male	18 (81.8)	7 (77.8)	11 (84.6)	0.683
Female	4 (18.2)	2 (22.2)	2 (15.4)	
**Occupation; *n* (%)**				
Driver	6 (27.3)	3 (33.3)	3 (23.1)	0.914
Employee/Worker	4 (18.2)	1 (11.1)	3 (23.1)	
Farmer	6 (27.3)	2 (22.2)	4 (30.8)	
Housewife	4 (18.2)	2 (22.2)	2 (15.4)	
Other	2 (9.1)	1 (11.1)	1 (7.7)	
**Skin type; *n* (%)**				
III	5 (22.7)	3 (33.3)	2 (15.4)	0.609
IV	17 (77.3)	6 (66.7)	11 (84.6)	
**Underlying diseases** ^**a**^ **; *n* (%)**				
Dyslipidemia	3 (13.6)	3 (33.3)	0 (0)	0.055
Blood pleasure	8 (36.4)	4 (44.4)	4 (30.8)	0.662
Diabetes	5 (22.7)	2 (22.2)	3 (23.1)	0.962
Ischemic heart disease	3 (13.6)	0 (0)	3 (23.1)	0.121

^a^ Only positive cases of underlying diseases or receiving medication are considered.

**FIGURE 2:**
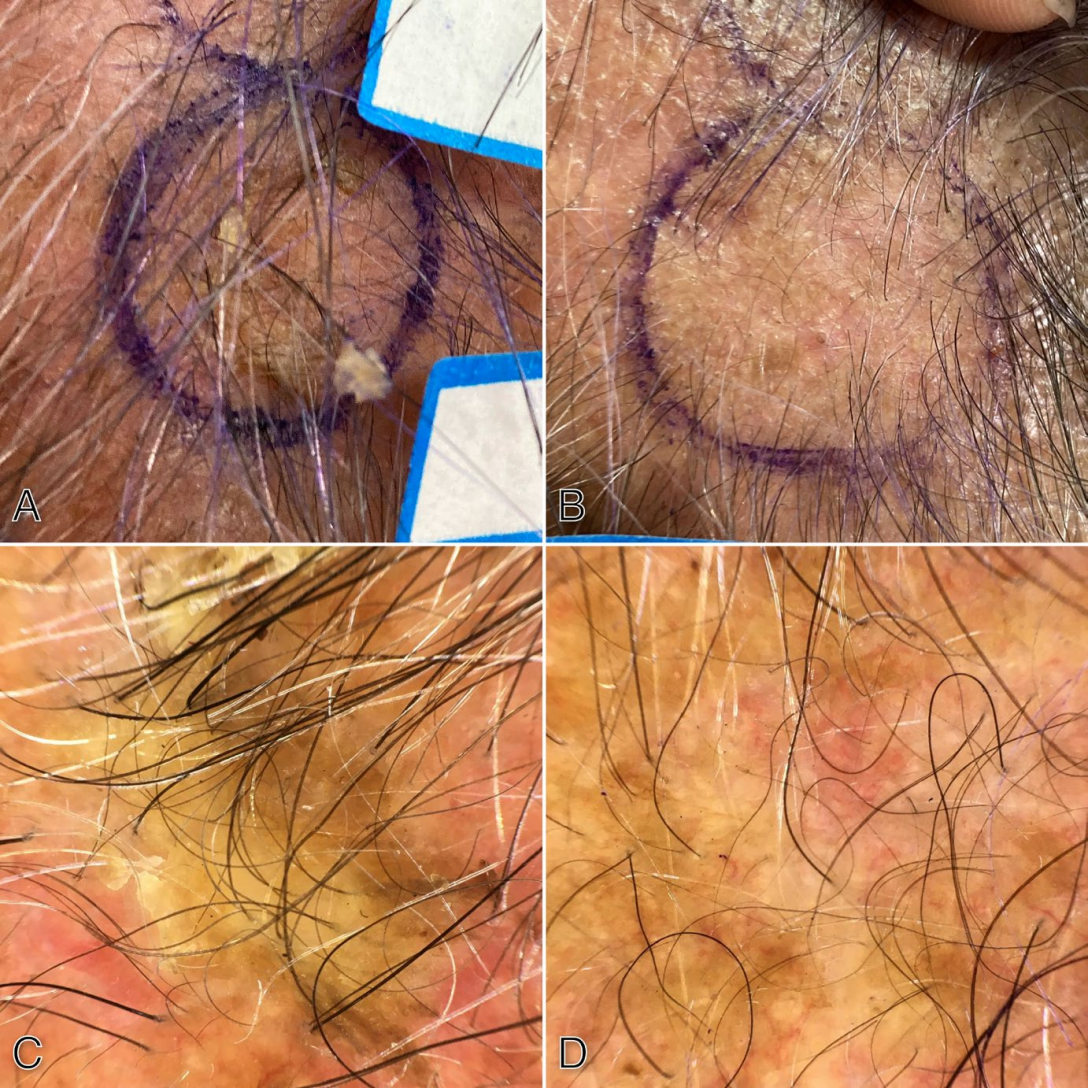
A 69‐year‐old man with AK of the scalp of before (A) and after (B) treatment with 5% fluorouracil topical cream. Dermoscopic pictures before treatment show thick white and yellow scales, jelly sign, and linear wavy vessels (C) which nearly completely have been resolved (D).

Based on the dermoscopic evaluation, in the fluorouracil treatment group, there was a significant decrease and improvement in dermoscopy grading score in rhomboidal pattern (*p* = 0.029), jelly sign (*p* = 0.037), star‐like appearance (*p* = 0.046), inner gray halo (*p* = 0.001), white circle sign (*p* = 0.002), rosette (*p* < 0.001), double white clod (*p* < 0.001), one white clod (*p* = 0.005), dermoscopic horn (*p* = 0.015), white scale (*p* < 0.001), yellow scale (*p* = 0.002) after treatment (Figure 2). However, no significant difference was observed in the annular granular pattern, red pseudo‐network, and linear wavy vessels compared to before treatment (Table 2).

**TABLE 2:** Comparison of changes in dermoscopic grading score over time between the two groups.

Dermoscopic grading	Time	Treatment		*p*
		Fluorouracil	Niacinamide	
Rhomboidal pattern	Pre‐treatment	0.174 ± 0.37	0.310 ± 0.46	0.166
	Post‐treatment	0.023 ± 0.15	0.143 ± 0.35	**0.046**
	Changes *p*‐value	**0.029**	**0.035**	**0.035**
Annular granular pattern	Pre‐treatment	0.186 ± 0.37	0.310 ± 0.45	0.185
	Post‐treatment	0.186 ± 0.37	0.167 ± 0.37	0.683
	Changes *p*‐value	0.890	**0.039**	**0.048**
Jelly sign	Pre‐treatment	0.814 ± 0.37	0.917 ± 0.26	0.145
	Post‐treatment	0.698 ± 0.80	0.786 ± 0.36	0.067
	Changes *p*‐value	**0.037**	**0.049**	0.264
Star like appearance	Pre‐treatment	0.093 ± 0.29	0.107 ± 0.3	0.723
	Post‐treatment	0.000 ± 0.00	0.036 ± 0.17	0.150
	Changes *p*‐value	**0.046**	0.187	0.469
Red pseudo‐network	Pre‐treatment	0.593 ± 0.49	0.619 ± 0.47	0.823
	Post‐treatment	0.453 ± 0.47	0.500 ± 0.45	0.632
	Changes *p*‐value	0.184	0.157	0.664
Inner Gray Halo	Pre‐treatment	0.465 ± 0.5	0.571 ± 0.5	0.330
	Post‐treatment	0.186 ± 0.37	0.738 ± 0.54	**< 0.001**
	Changes *p*‐value	**0.001**	0.052	**0.001**
White circle sign	Pre‐treatment	0.465 ± 0.5	0.476 ± 0.5	0.919
	Post‐treatment	0.163 ± 0.37	0.476 ± 0.5	**0.002**
	Changes *p*‐value	**0.002**	1.000	**0.042**
Rosette	Pre‐treatment	0.442 ± 0.5	0.238 ± 0.43	0.051
	Post‐treatment	0.116 ± 0.3	0.143 ± 0.35	0.896
	Changes *p*‐value	**< 0.001**	0.206	**0.021**
Double white clod	Pre‐treatment	0.581 ± 0.49	0.524 ± 0.5	0.596
	Post‐treatment	0.070 ± 0.25	0.619 ± 0.49	**< 0.001**
	Changes *p*‐value	**< 0.001**	0.285	**0.002**
One white clod	Pre‐treatment	0.721 ± 0.45	0.762 ± 0.43	0.668
	Post‐treatment	0.465 ± 0.49	0.762 ± 0.43	**0.004**
	Changes *p*‐value	**0.005**	1.000	**0.034**
Dermoscopic horn	Pre‐treatment	0.140 ± 0.356	0.369 ± 0.53	**0.021**
	Post‐treatment	0.047 ± 0.21	0.321 ± 0.46	**0.001**
	Changes *p*‐value	0.015	0.651	**0.001**
White scale	Pre‐treatment	0.709 ± 0.53	0.699 ± 0.59	0.562
	Post‐treatment	0.140 ± 0.33	0.534 ± 0.49	**< 0.001**
	Changes *p*‐value	**< 0.001**	0.189	**0.046**
Yellow scale	Pre‐treatment	0.465 ± 0.66	0.429 ± 0.66	0.747
	Post‐treatment	0.093 ± 0.27	0.417 ± 0.62	**0.005**
	Changes *p*‐value	**0.002**	0.857	0.160
Linear wavy vessels	Pre‐treatment	0.721 ± 0.39	0.750 ± 0.48	0.589
	Post‐treatment	0.733 ± 0.38	0.679 ± 0.47	0.534
	Changes *p*‐value	0.913	0.141	0.878

*Note:* Values are reported as mean ± standard deviation or as *p*‐values. Bold values means there is statistically significant difference between the 2 groups (first column for Fluorouracil and the second column for Niacinamide).

**FIGURE 3:**
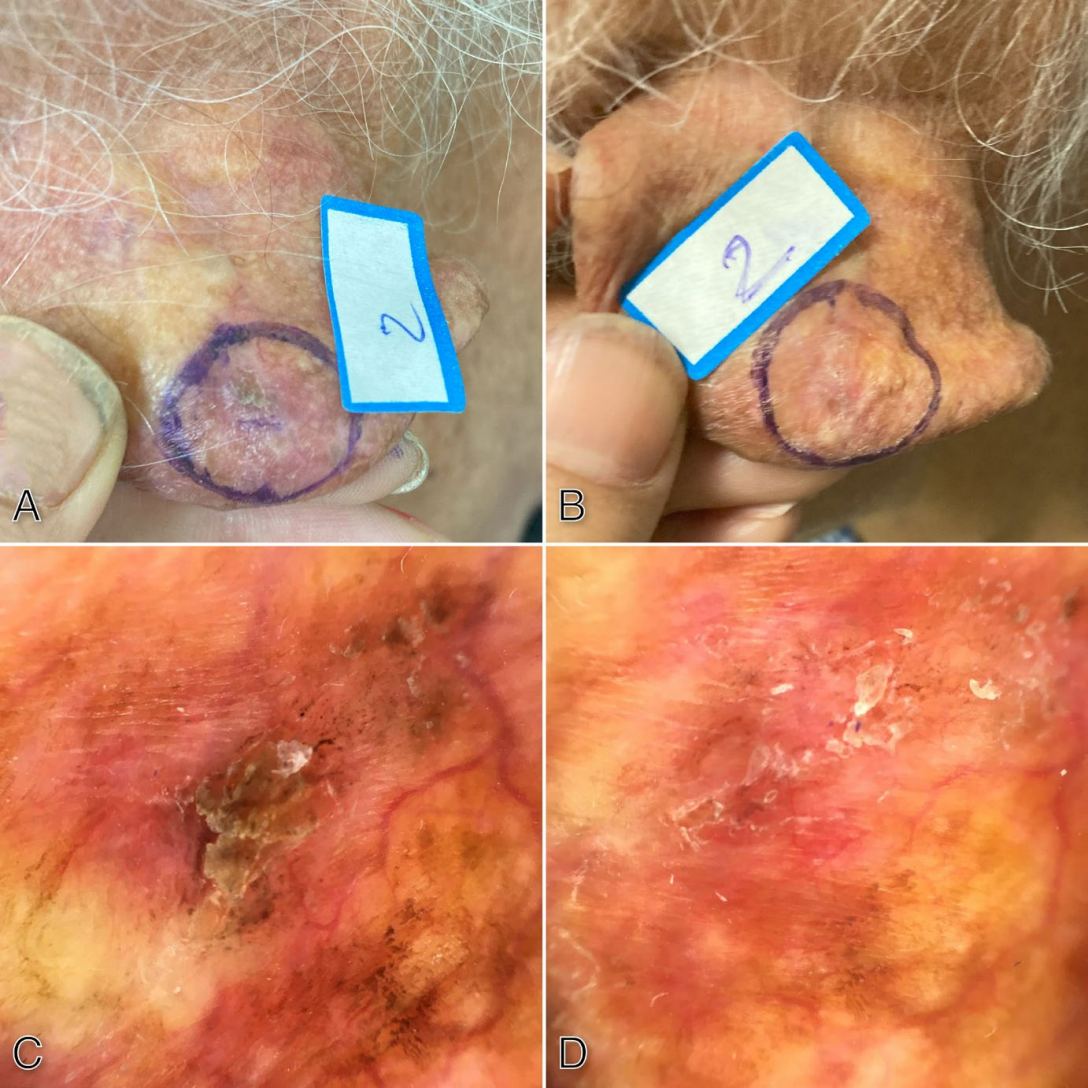
A 76‐year‐old woman with AK behind the ear before (A) and three months after (B) treatment with 1% niacinamide. Dermoscopic features before treatment demonstrate annular granular pattern, jelly sign, thick white and yellow scale, and wavy vessels (C) which significantly resolved after treatment (D).

Dermoscopic evaluation of the niacinamide treatment group showed a significant decrease in the grading score of the rhomboidal pattern (*p* = 0.035), annular granular pattern (*p* = 0.039), and jelly sign (*p* = 0.037) (Figure 3). However, in dermoscopic signs including star‐like appearance, inner gray halo, white circle sign, rosette, double white clod, one white clod, dermoscopic horn, white scale, yellow scale, red pseudo‐network, and linear wavy vessels, no significant difference was observed compared to before treatment (Table 2).

The comparison of dermoscopy grading factors between the two groups after treatment compared to the baseline showed that rhomboidal pattern (*p* = 0.035), inner gray halo (*p* = 0.001), white circle sign (*p* = 0.042), rosette (*p* = 0.021), double white clod (*p* = 0.002), one white clod (*p* = 0.034), dermoscopic horn (*p* = 0.001) and white scale (*p* = 0.046) were decreased more significantly in the fluorouracil group than in the niacinamide group. However, the annular granular pattern significantly decreased more in the niacinamide group (*p* = 0.048) than in the fluorouracil group (Table 2).

Evaluation of photographic and dermoscopic pictures by two blind dermatologists showed more significant improvement in the 5FU group than in the niacinamide group. In addition, evaluating patient satisfaction with their treatment revealed patients were significantly more satisfied with 5FU (Table 3).

**TABLE 3:** Photographic and dermoscopic assessment of the two groups.

Method of improvement assessment	Treatment		*p*
	Fluorouracil	Niacinamide	
**Photography score improvement, *n* (%)**			
0%–25%	7 (16.3)	20 (46.7)	**0.010**
25%–50%	9 (20.9)	9 (21.4)	
50%–75%	10 (23.3)	6 (14.3)	
75%–100%	17 (39.5)	7 (16.7)	
**Dermoscopy score improvement, *n* (%)**			
0%–25%	8 (18.6)	19 (45.2)	**0.011**
25%–50%	9 (20.9)	12 (28.6)	
50%–75%	14 (32.6)	5 (11.9)	
75%–100%	12 (27.9)	6 (14.3)	
Patient's satisfaction score, mean ± standard deviation	7.97 ± 1.73	5.16 ± 2.91	**< 0.001**

*Note:* Bold values means there is statistically significant difference between the 2 groups (first column for Fluorouracil and the second column for Niacinamide).

Overall, no serious adverse effects were reported during the trial. However, local reactions including erythema, burning sensation, and itching were observed more significantly in the 5FU group (Table 4).

**TABLE 4:** Comparison of side effects between the two groups.

Side effects	Severity	Fluorouracil *F* (%)	*Niacinamide n (%)*	*p*
Burning, *n* (%)	None	27 (62.8)	41 (97.6)	**0.001**
	Mild	9 (20.9)	1 (2.4)	
	Moderate	1 (2.3)	0 (0)	
	Severe	6 (14)	0 (0)	
Itching, *n* (%)	None	34 (79.1)	42 (100)	**0.002**
	Mild	9 (20.9)	0 (0)	
Erythema, *n* (%)	None	12 (27.9)	38 (90.5)	**< 0.001**
	Mild	22 (51.2)	4 (9.5)	
	Moderate	3 (7)	0 (0)	
	Severe	6 (14)	0 (0)	

*Note:* Bold values means there is statistically significant difference between the 2 groups (first column for Fluorouracil and the second column for Niacinamide).

## 4 Discussion

In this study, we demonstrated the higher therapeutic effect of 5% fluorouracil cream compared to 1% niacinamide gel in treating AK lesions. In the fluorouracil group, 37.2% of lesions have mild to moderate improvement and 62.8% have good‐to‐excellent improvement, while in the niacinamide group, 68.1% of the lesions have mild to moderate improvement and 31% have good to excellent improvement. On the other hand, the adverse effects included burning (37.2% vs. 2.4%), erythema (72.2% vs. 9.5%), and itching (20.9% vs. 0) was significantly more common in the 5‐FU group. These results demonstrate that although fluorouracil is more effective in treating AK, it should be prescribed with caution.

Our data demonstrated that fluorouracil outperformed niacinamide in terms of dermoscopic features including rhomboidal pattern, inner gray halo, white circle sign, rosette, double white clod, one white clod, dermoscopic horn, and white scale, but not the annular granular pattern. To date, there have been limited reports comparing and evaluating dermoscopic treatment responses for actinic keratosis. Salehi Farid et al. reported a dermoscopic response to 5% fluorouracil treatment in 46% of AK patients after the first month, and 59% after the third month. The main improved dermoscopic features included yellow scale, white scale, and pseudo‐network pattern; however, no significant change was observed in pigmented dots and linear‐wavy vessels. The effect of topical treatment varies based on the penetration into the dermis, and may not always affect features related to the deep dermis (e.g., linear wavy vessels), rather than the superficial layer (e.g., white and yellow scales). Furthermore, there was a significant direct correlation between dermoscopic response rates and clinical response [14].

Fluorouracil 5% is a pyrimidine analog, belonging to the family of antimetabolites. Its mechanism of action is based on the inhibition of thymidylate synthase, an enzyme needed for DNA synthesis and the formation and function of RNA. This results in the destruction of the precancerous cells while sparing normal skin cells [2, 3]. Additionally, it causes the release of cell antigens that heighten the immune system reaction, achieving an inflammatory response. This action aids in its necrotic abilities and leads to AK clearance [15]. Various formulations of the drug are commercially available, including 5% cream or solution, 1% cream or solution, 2% solution, and 0.5% cream, that can be applied once or twice daily in the area of AK lesions for 2–4 weeks up to a maximum of 12 weeks [2, 16]. Previous studies have shown that Olsen grades I and II of actinic keratosis of the face, ears, and/or scalp in adults are suitable for topical treatment with 5‐FU 4% cream. This medication was approved based on results from two controlled phases III primary efficacy studies demonstrating that the application of 5‐FU 4% cream once daily compared to twice daily results in similar rates of partial and complete clearance (54.4% vs. 57.9% and 80.5% vs. 80.2%, respectively) of AK lesions [16]. Fluorouracil identified led to a range of 42.9%–76.6% reductions of AK counts at 12‐month follow‐up [17, 18, 19, 20].

Niacinamide (vitamin B3) is the precursor for niacinamide adenine dinucleotide, which is central to cellular energy and DNA repair [21]. Niacinamide normalizes energy production genes and enzymes in UV‐exposed keratinocytes, which are adenosine triphosphate deficient [22, 23, 24]. In mice, 2.5% niacinamide reduced UV immunosuppression and photocarcinogenesis [25]. However, in a meta‐analysis by Mainville et al. [15] niacinamide demonstrated no significant effect on the treatment of AK, their study mainly focused on the oral form of the medication and reported a heterogenicity among the published literature. Our data also demonstrated that most patients in the niacinamide group demonstrated less than 50% improvement in their treatment. Topical niacinamide showed a significant reduction in AKs compared to vehicle [25]. Although there are limited studies evaluating the efficacy and safety of topical niacinamide in the treatment of AK, its pharmacological properties, low side effects, and relative improvement suggest that it could be considered as an adjunctive and complementary treatment for AK, particularly when used in conjunction with 5‐FU.

Limitations of our study include the small sample size, single‐center design, lack of histological evaluation, absence of long‐term patient follow‐up and recurrence rate assessment, and variations in treatment duration based on medication mechanism and assigned regimen in our study.

## 5 Conclusion

This present study indicates that 5% fluorouracil cream is more effective than 1% niacinamide gel in the treatment of AK lesions based on clinical and dermoscopic evaluation, however accompanied by more side effects. Finally, studies with larger sample sizes and longer follow‐ups are recommended.

## Author Contributions

N.P., M.S., and M.B. performed the research. N.P. and M.B. designed the research study. M.B. analyzed the data and N.P. wrote the paper. M.S. and Z.S. edit the paper.

## Ethics Statement

The study was approved by Isfahan University's Institutional Review Board and Ethics Committee (IR.MUI.MED.REC.1400.445) and was registered in the Iranian Registry of Clinical Trial (IRCT20220519054920N1).

## Conflicts of Interest

The authors declare no conflicts of interest.

## Data Availability

The data that support the findings of this study are available on request from the corresponding author. The data are not publicly available due to privacy or ethical restrictions.
